# The c-Myc-regulated lncRNA NEAT1 and paraspeckles modulate imatinib-induced apoptosis in CML cells

**DOI:** 10.1186/s12943-018-0884-z

**Published:** 2018-08-28

**Authors:** Chengwu Zeng, Sichu Liu, Shuai Lu, Xibao Yu, Jing Lai, Yifan Wu, Shaohua Chen, Liang Wang, Zhi Yu, Gengxin Luo, Yangqiu Li

**Affiliations:** 1 Key Laboratory for Regenerative Medicine of Ministry of Education, Institute of Hematology, Jinan University, Guangzhou, 510632 China; 2 Department of Hematology, First Affiliated Hospital, Jinan University, Guangzhou, 510632 China; 3 Department of Oncology, First Hospital Affiliated, Jinan University, Guangzhou, 510632 China

**Keywords:** CML, LncRNA NEAT1, SFPQ, IM, Paraspeckle, Apoptosis

## Abstract

Chronic myeloid leukemia (CML) is a clonal disease characterized by the presence of the constitutively active tyrosine kinase BCR-ABL oncoprotein. Although BCR-ABL is crucially important for pathogenesis and treatment response, it is thought that some additional factors might be involved in the regulation of these processes. Aberrant expression of long noncoding RNAs (lncRNAs) has recently been identified to be involved in various diseases including cancer, suggesting that lncRNAs may play a role in BCR-ABL-mediated CML. In this study, we found that nuclear-enriched abundant transcript 1 (NEAT1), a lncRNA essential for the formation of nuclear body paraspeckles, is significantly repressed in primary CML cells. NEAT1 expression could be restored by inhibiting BCR-ABL expression or its kinase activity in K562 cells. We also demonstrated that NEAT1 is regulated by c-Myc. Knockdown of NEAT1 could promote imatinib (IM)-induced apoptosis, and we demonstrated that the NEAT1-binding paraspeckle protein splicing factor proline/glutamine-rich (SFPQ) is required for NEAT1-mediated apoptosis in K562 cells. RNA-seq analysis revealed that SFPQ regulates cell growth and death pathway-related genes, confirming its function in IM-induced apoptosis. Collectively, these results assign a biological function to the NEAT1 lncRNA in CML apoptosis and may lead to fuller understanding of the molecular events leading to CML.

**Electronic supplementary material:**

The online version of this article (10.1186/s12943-018-0884-z) contains supplementary material, which is available to authorized users.

## Main text

Chronic myelogenous leukemia (CML) is a malignant, clonal hematopoietic stem cell disorder characterized by a reciprocal translocation between chromosomes 9 and 22 [1]. This translocation results in the formation of the BCR-ABL oncogene, which encodes a chimeric BCR-ABL protein with constitutively active tyrosine kinase activity [2]. Imatinib (IM) was the first BCR–ABL tyrosine kinase inhibitor (TKI) and a frontline drug used in CML therapy. IM binds the ATP-binding site of BCR-ABL, thus preventing a conformational switch to the active form of the oncoprotein, blocking its downstream signal transduction.

Compared with the research progress of microRNAs, there are thousands of longer transcripts, long non-coding RNAs (lncRNAs), whose functions are unknown [3]. We previously demonstrated that the lncRNA NEAT1 (nuclear paraspeckle assembly transcript 1) plays a significant role in the regulation of acute promyelocytic leukemia (APL) cell differentiation [4]. NEAT1, which was demonstrated to be an essential structural component of the subnuclear structure paraspeckle, has two isoforms: 3.7 kb NEAT1_1 and 23 kb NEAT1_2. Recent studies have demonstrated that NEAT1 plays a crucial role in cellular transformation [5]. Despite the importance of NEAT1 in various cancers, little is known about the precise function and mechanism of regulation of nuclear paraspeckles. In addition, the involvement of NEAT1 in CML has not been defined, and the mechanism by which NEAT1 functions to impact BCR-ABL-induced tumorigenesis is unknown.

## LncRNA NEAT1 is downregulated by BCR-ABL

**Fig. 1:**
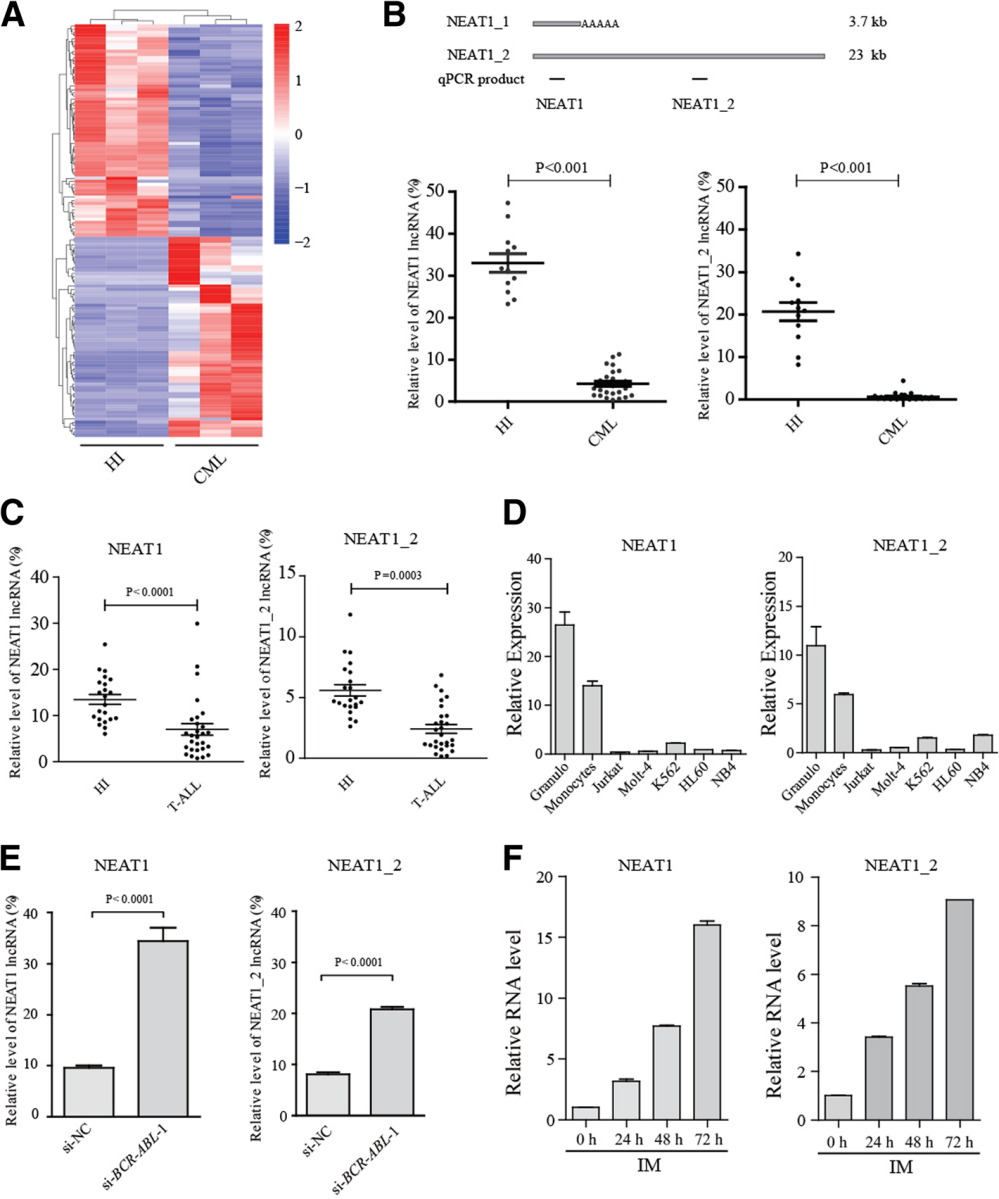
The lncRNA NEAT1 is significantly down-regulated in CML **a** Supervised hierarchical clustering analysis of 377 lncRNAs that were consistently upregulated or downregulated in CML samples (≥ 2-fold and FDR ≤ 0.001). The red and blue shading is used to illustrate whether the expression values are above (red) or below (blue) the mean expression value across all samples. **b** The NEAT1 isoforms are shown schematically (up panel). The black boxes indicate the position of the sequences amplified by qRT-PCR. Comparison of NEAT1 expression in granulocytes from healthy donors (Granulo, *n* = 12) compared with primary CML cells (*n* = 26) (lower panel). The values were normalized to the expression level of the housekeeping gene *ACTB*. Each data point represents 1 patient sample. **c** Comparison of NEAT1 expression in PBMCs from healthy donors (*n* = 21) compared with primary T-ALL cells (*n* = 28). The expression levels of the NEAT1 isoforms were evaluated by qRT-PCR. **d** Expression of NEAT1 in K562, NB4, HL60, Jurkat, and Molt4 cells. **e** qRT–PCR analysis of NEAT1 after BCR-ABL knockdown. **f** qRT–PCR analysis of NEAT1 in K562 cells treated with 1 μM IM for the indicated times

To identify lncRNAs associated with CML, we began by comparing the transcriptomes of peripheral blood obtained from patients with CML and healthy donors. RNA sequencing data processing and assessment of the transcriptome were conducted as detailed in our previous study [6]. The RNA-seq data analysis revealed 200 upregulated lncRNAs and 177 downregulated lncRNAs in CML patients (Fig. 1a and Additional file 1: Table S1). We selected several differentially expressed, well-studied lncRNAs for validation by qRT-PCR (Additional file 2: Figure S1A). From these analyses, we found that the lncRNA NEAT1 was downregulated in CML. Next, we examined the NEAT1 expression level in peripheral blood mononuclear cells (PBMCs) from 26 cases with de novo CML. qRT-PCR revealed that both NEAT1 and NEAT1_2 were significantly decreased in CML patient samples compared with granulocytes from healthy individuals (Fig. 1b). A similar pattern of expression was also observed in T-ALL (T cell acute lymphoblastic leukemia) patients (Fig. 1c) and several leukemia cell lines (Fig. 1d). This result suggested that NEAT1 may be involved in CML pathogenesis.

The BCR-ABL fusion protein is known to be the initiating factor for CML development via multiple pathways; thus, we explored whether NEAT1 downregulation was a consequence of BCR-ABL expression. We found that silencing BCR-ABL expression markedly increased the expression of NEAT1 (total NEAT1 and NEAT1_2) in CML cell line K562 cells (Fig. 1e and Additional file 2: Figure S1B). Additionally, we found that IM treatment could enhance NEAT1 expression in K562 cells (Fig. 1f). Importantly, this effect was also observed in primary CML cells that were freshly isolated from CML patients (Additional file 2: Figure S1C). The expression of NEAT1 could be restored after treatment with inhibitors of BCR-ABL-mediated pathways or repressed by an activator of a BCR-ABL-mediated pathway (Additional file 2: Figure S1D), suggesting that enhanced NEAT1 expression may depend on BCR-ABL-mediated pathways.

## NEAT1 is transcriptionally regulated by c-Myc

**Fig. 2:**
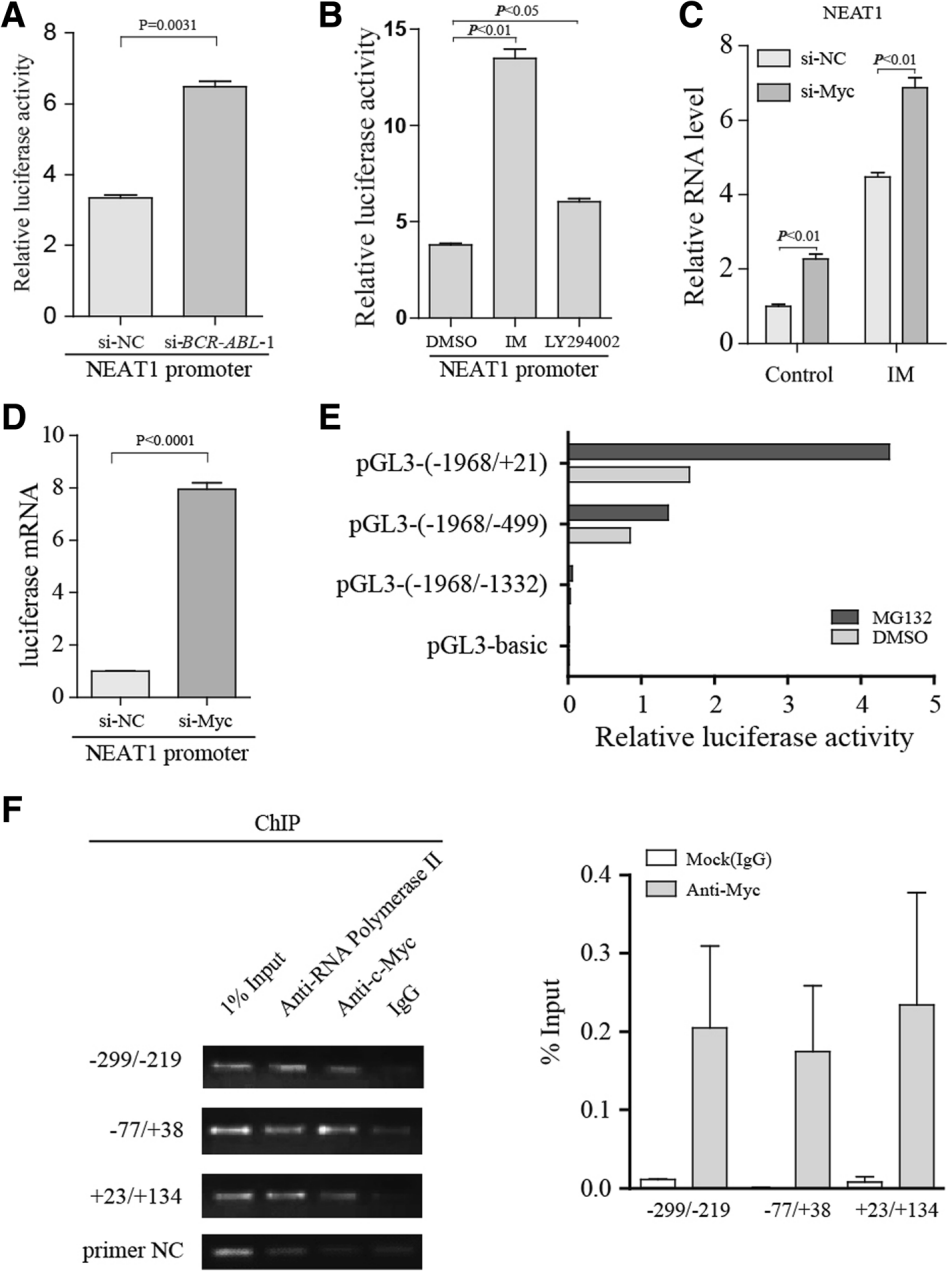
NEAT1 is regulated by c-Myc in K562 cells. **a** K562 cells were transfected with si-BCR-ABL and NEAT1 promoter constructs. Twenty-four hours after transfection, the luciferase activity was analyzed. **b** Luciferase reporter assays were performed with K562 cells using the indicated reporters, and the cells were treated with LY204002 and IM. **c** qPCR analysis of the expression of total NEAT1 in K562 cells following transfection with control siRNA and c-Myc siRNA, demonstrating marked dependence on c-Myc. **d** Luciferase reporter assays were performed with cells transfected with NEAT1 promoter constructs together with si-Myc or control siRNA. **e** Cells were transfected with NEAT1 promoter constructs (Renilla luciferase reporter construct as a control vector) and treated with DMSO or MG132 (Previous studies have demonstrated that NEAT1 is transcriptionally up-regulated by the proteasome inhibitor MG132 in Hela cells), and luciferase activity was measured after 24 h. **e** c-Myc ChIP followed by qPCR analyzed the occupation of c-Myc on the NEAT1 gene. The co-immunoprecipitated DNA was amplified by PCR using primer pairs spanning a 500 bp region. Primers that amplify non-c-Myc binding regions were used as negative control(primer NC). The data are shown as a percentage of the input

Since NEAT1 is regulated by BCR-ABL-mediated pathways, the luciferase activity of the NEAT1 promoter (methods see Additional file 3) was significantly activated by si-BCR-ABL treatment (Fig. 2a), and it was also activated by inhibitors of BCR-ABL-mediated pathway (Fig. 2b). These results suggest that NEAT1 might be transcriptionally regulated by a downstream effector of BCR-ABL-mediated pathways. NEAT1 has already been identified as a p53 target [7]. However, unlike most cancer types, the frequency of p53 mutations in CML is low [8]. c-Myc was previously identified as a common regulator of pathways downstream of BCR-ABL [9]; thus, we hypothesized that c-Myc might regulate NEAT1 expression. As expected, we found that NEAT1 expression was increased by c-Myc knockdown, and treatment of the c-Myc knockdown cells with IM further increased NEAT1 expression (Fig. 2c). Consistent with previous reports, the c-Myc target gene miR-17HG and p27 were regulated by c-Myc knockdown (Additional file 2: Figure S2A). Importantly, NEAT1 promoter activity was induced by transfection with si-Myc (Fig. 2d). Deletion analysis demonstrated that promoter activity is retained in the ~ 500 bp region upstream of the transcription start site (Fig. 2e). To test whether c-Myc could interact with the endogenous NEAT1 promoter, a ChIP assay was performed using K562 cells. Primers flanking the 500 bp region upstream and the transcriptional start site were then used in qRT-PCR assays in which we could detect c-Myc binding at the NEAT1 promoter (Fig. 2f). Collectively, our results demonstrated that c-Myc can act directly with the NEAT1 promoter in CML cells.

## NEAT1 inhibition enhances IM-induced apoptosis and is linked to paraspeckle protein

**Fig. 3:**
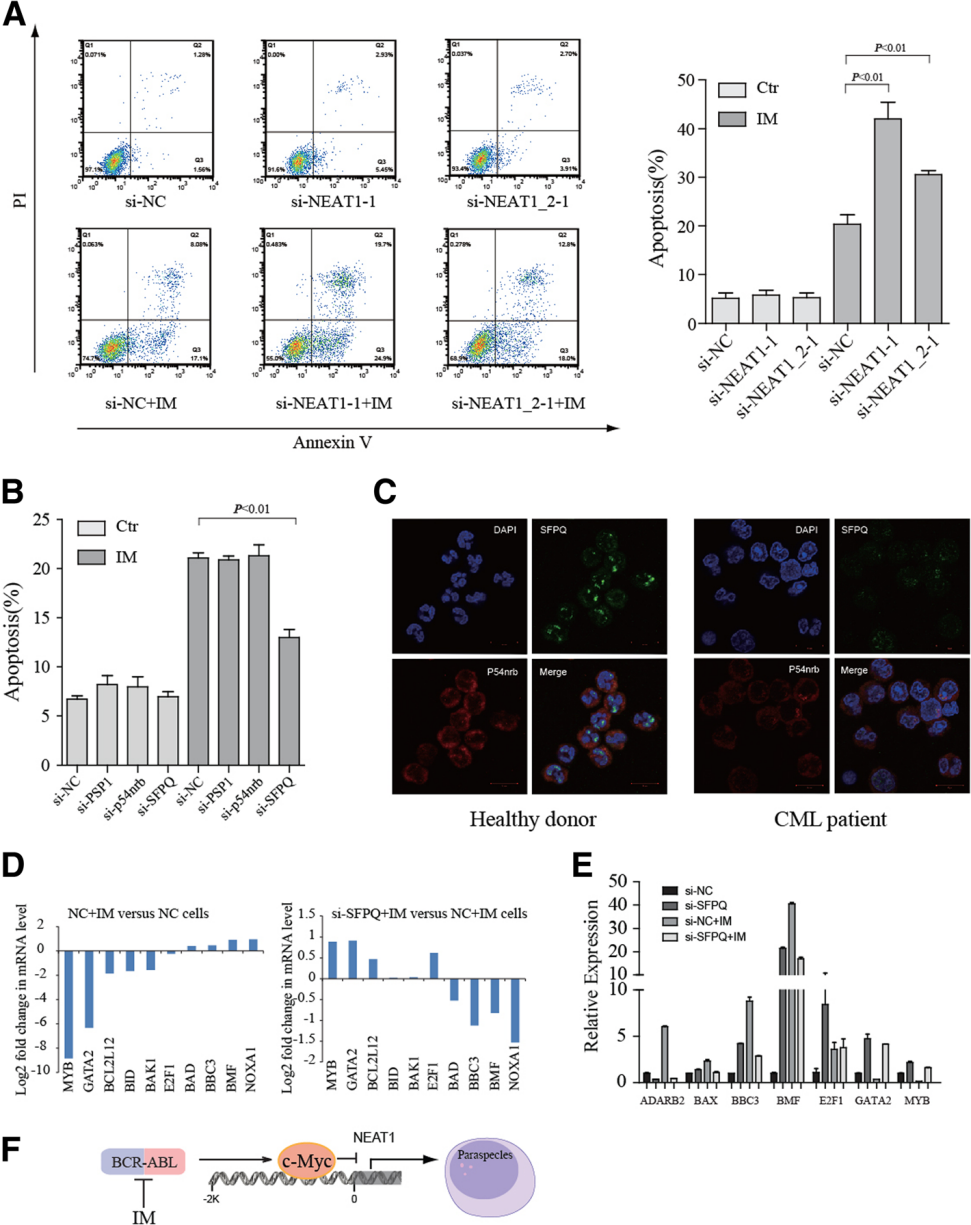
NEAT1 paraspeckles modulate imatinib-induced apoptosis in K562 cells. **a** K562 cells were transfected with control siRNA or NEAT1 siRNA followed by a 48 h IM treatment. Apoptosis was assessed by annexin V and propidium iodide staining, and apoptotic cells were identified by flow cytometry. Data are shown as the mean ± SD of three separate experiments. **b** Identification of the paraspeckle proteins involved in IM-induced apoptosis. K562 cells were transfected with control siRNA or paraspeckle proteins siRNA followed by a 48 h treatment with IM. Apoptosis was analysis by flow cytometry, and values were normalized to untreated control cells. **c** The cells were visualized by confocal microscopy for SFPQ (green) and p54nrb (red). DAPI was used to indicate DNA. Cells were stained with anti-p54nrb and anti-SFPQ antibodies. **d** SFPQ knockdown in K562 cells repressed the modification of a large number of Bcl2 family genes in response to IM stimuli (see Additional file 4: Table S2 for expression profiling of all genes whose expression is specifically altered in K562 cells). **e** qRT–PCR analysis of gene expression to verify the accuracy of RNA-seq. **f** Model of Myc-mediated NEAT1 expression. BCR-ABL-activated pathways increase c-Myc expression, c-Myc then directly interacts with the NEAT1 promter, repressing NEAT1 transcription. NEAT1 lncRNA expression dictates paraspeckle size and shape. Downregulated-NEAT1 frees the paraspeckle proteins such as SFPQ from paraspeckles

We next explored the functional role of NEAT1 in the IM-induced apoptosis in CML cells. NEAT1 was shown to be effectively knocked down by siRNA (Additional file 2: Figure S3A and S3B). The results demonstrated that NEAT1 knockdown combined with IM treatment could promote the apoptosis of K562 cells (Fig. 3a and Additional file 2: Figure S3C). Similarly, NEAT1-knockdown CML cells were more sensitive to proteasome inhibition (Additional file 2: Figure S3D). NEAT1 has been reported to be critical for nuclear body paraspeckle formation. It is known that SFPQ (splicing factor proline/glutamine-rich), NONO (p54nrb), and PSPC1 (paraspeckle component 1) are protein components of paraspeckles [10]. We found that siRNA-mediated depletion of SFPQ, but not p54nrb or PSPC1, prevented the apoptotic effects induced by IM in K562 cells (Fig. 3b). Accordingly, specific depletion of SFPQ displayed a less severe effect compared with control cells in proteasome inhibitor MG132-induced apoptosis (Additional file 2: Figure S3E). These results suggested that the SFPQ is required for NEAT1 mediated-apoptosis. Strikingly, we observed that the distribution of SFPQ protein in the nucleus was altered. SFPQ adopted a more punctate distribution in normal granulocytes (Fig. 3c), indicating decreased formation of nuclear paraspeckles in CML cells. These data suggested that NEAT1 may sequester SFPQ within paraspeckles, thereby limiting the availability of SFPQ to promote apoptosis [10].

To reveal the downstream targets of SFPQ, we analyzed differential gene expression using RNA-seq data from SFPQ-knockdown cells treated with or without IM (Additional file 2: Figure S3F and Additional file 4: Table S2). Of note, pathway analysis using DAVID Bioinformatics revealed that cell growth and death pathway genes were modulated by IM but resisted in the presence of si-SFPQ. (Fig. 3d and Additional file 5: Table S3), which is consistent with our observation that SFPQ participates in IM-induced apoptosis. Validation by quantitative real-time PCR demonstrated that SFPQ knockdown significantly reduces the expression of Bcl-2 family pro-apoptosis genes such as BBC3, BAX and BMF in IM-induced apoptosis (Fig. 3e). Our findings suggested that SFPQ regulates IM-induced apoptosis via cell death pathway proteins. Taken together, our findings that inactivation of c-Myc regulates NEAT1 expression, and NEAT1 paraspeckles, in turn, attenuated IM-induced apoptosis, suggesting an autoregulatory negative feedback loop that exerts c-Myc activity in apoptosis (Fig. 3f).

## Conclusion

In summary, we showed that NEAT1 is an essential mediator of the apoptosis induced by IM in BCR-ABL-expressing cells. Moreover, we demonstrated that NEAT1 is a direct transcriptional target of c-Myc, c-Myc represses NEAT1 expression by binding to the NEAT1 promoter, and SFPQ is required for NEAT1-mediated apoptosis in K562 cells. Together, these findings suggest that targeting NEAT1 might represent a novel and important therapeutic strategy for treating leukemia.

## Additional files

Additional file 1: **Table S1.** LncRNA gene expression level in CML and the healthy donor. (XLSX 43 kb)

Additional file 2: **Figure S1.** Analysis of lncRNA expression. **Figure S2.** Imatinib-induced NEAT1 is associated with c-Myc. **Figure S3.** Effects of NEAT1 on apoptosis in K562 cells. (DOCX 524 kb)

Additional file 3: Materials and methods. (DOCX 19 kb)

Additional file 4: **Table S2.** Gene expression level in si-NC and si-SFPQ cells with or without IM treatment. (XLSX 1424 kb)

Additional file 5: **Table S3.** Pathway analysis of genes regulated by SFPQ upon IM treatment. (XLSX 54 kb)

## Abbreviations

ChIP: Chromatin immunoprecipitation
CML: Chronic myeloid leukemia
IM: Imatinib
LncRNA: Long noncoding RNA
NEAT1: Nuclear-enriched abundant transcript 1
SFPQ: Splicing factor proline/glutamine-rich
T-ALL: T cell acute lymphoblastic leukemia
