# Supplementary material for: The c-Myc-regulated lncRNA NEAT1 and paraspeckles modulate imatinib-induced apoptosis in CML cells
Source: Mol Cancer. 2018 Aug 28;17:130. doi: 10.1186/s12943-018-0884-z (PMC6114538; doi:10.1186/s12943-018-0884-z)
Supplement: Supplementary file 2 — Figure S1. Analysis of lncRNA expression. Figure S2. Imatinib-induced NEAT1 is associated with c-Myc. Figure S3. Effects of NEAT1 on apoptosis in K562 cells. (DOCX 524 kb) [file 12943_2018_884_MOESM2_ESM.docx]

**Additional file 2:**


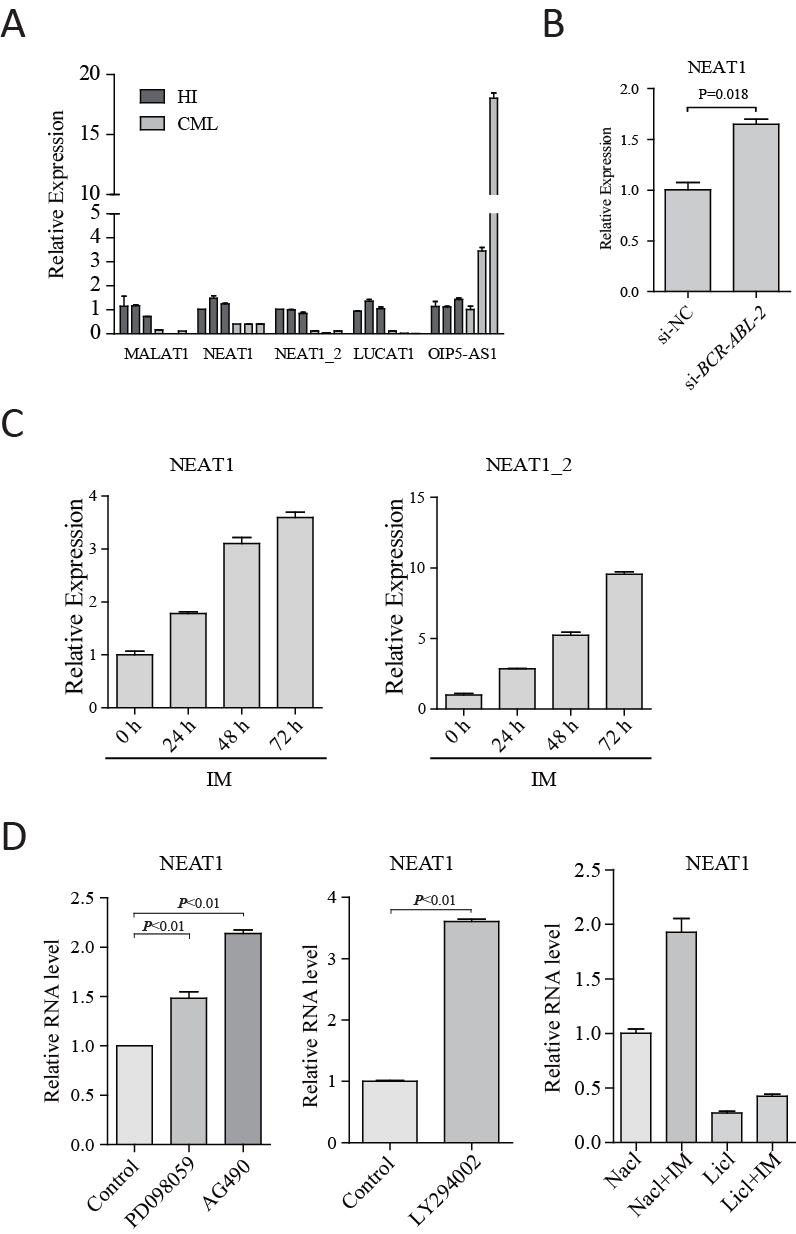


**Figure S1.** Analysis of lncRNA expression. (A) qRT–PCR analysis of lncRNA gene expression to verify the accuracy of RNA-seq. (B) qRT–PCR analysis of NEAT1 after BCR-ABL knockdown. (C) IM treatment results in elevated NEAT1 in CML cells. Tumor cells were obtained from patients with CML at diagnosis. The cells were then treated with 1 μM IM for the indicated time. A representative experiment demonstrating the NEAT1 levels as measured by qRT–PCR is shown. (D) K562 cells were treated with the MAPK inhibitor PD098059, the PI3K inhibitor LY204002, the JAK/STAT inhibitor AG490, and the Wnt pathway activator LiCl. Inhibition of BCR-ABL-mediated pathways resulted in elevation of NEAT1 lncRNA, and NEAT1 was repressed by the Wnt activator. NEAT1 was measured by qRT-PCR and normalized to the housekeeping gene *ACTB*.


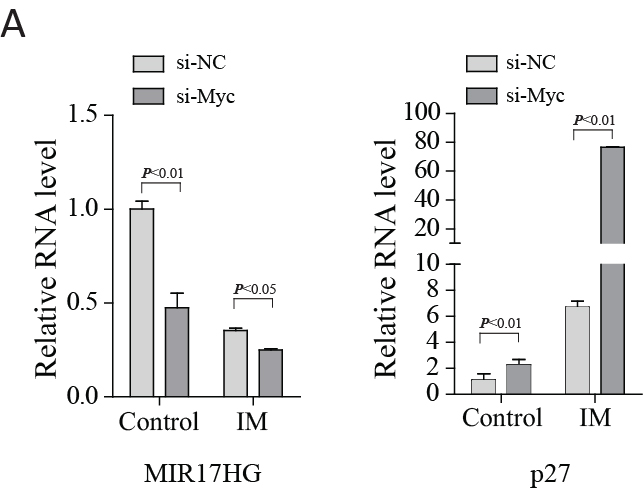


**Figure S2.** Imatinib-induced NEAT1 is associated with c-Myc. (A) K562 cells transfected with si-Myc showed a decrease in the expression of miR-17HG and an increase in expression of p27 compared with si-NC.

**
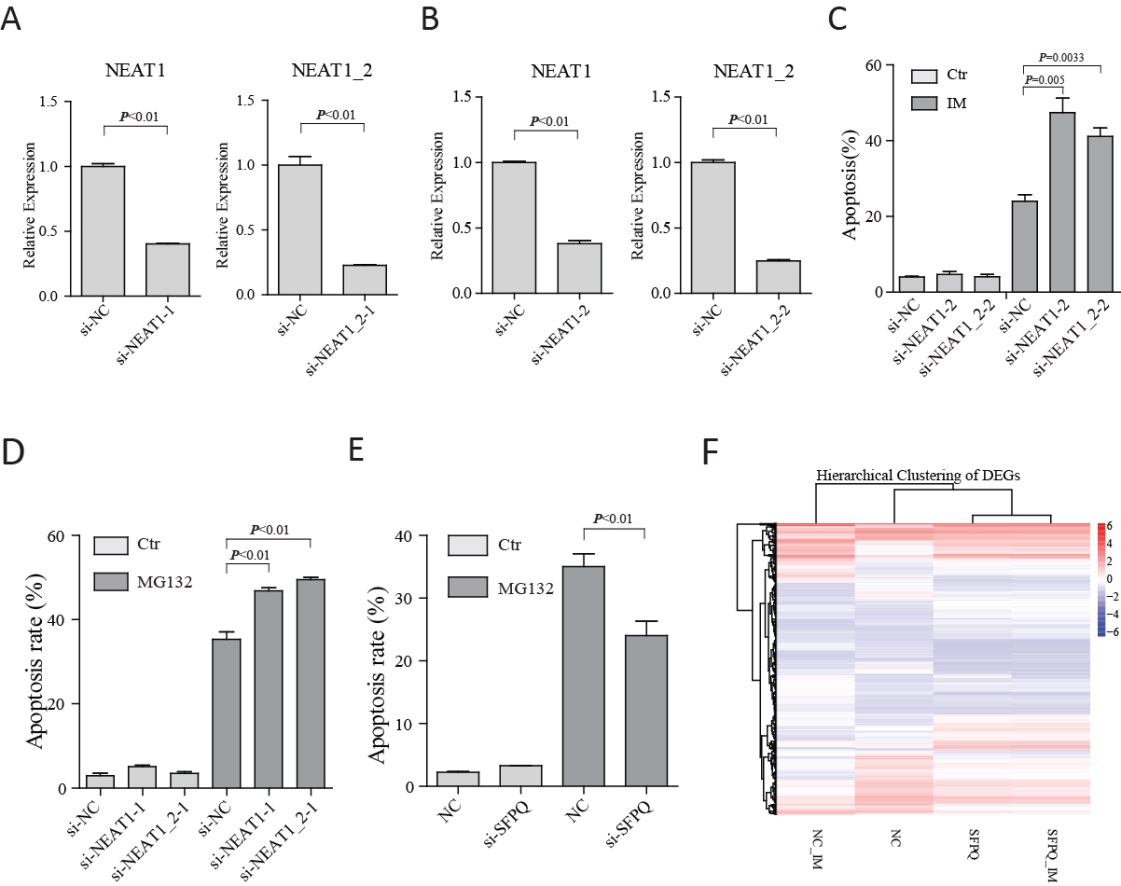
**

**Figure S3.** Effects of NEAT1 on apoptosis in K562 cells. (A and B) 48 hrs after transfection, the knockdown efficiency was confirmed by qRT-PCR. (C) K562 cells were transfected with control siRNA or the second NEAT1 siRNA followed by a 48 h IM treatment. Apoptosis was assessed by flow cytometry. (D) K562 cells were transfected with si-NC or si-NEAT1 followed by 36 h treatment with MG132 (1 μM). (E) Flow cytometry analysis evaluating the apoptosis of control and si-SFPQ cells after 36 h of MG132 (1 μM). (F) Heatmap displaying the hierarchical clustering of genes differentially expressed in si-NC and si-SFPQ cells in response to IM treatment. The color intensities represent the degree of expression. A red-blue color scale was used to reflect a standardization of gene expression with red representing high expression and blue representing low expression (scale shown in the upper left). A FDR cutoff of ≤ 0.001 and a log2-fold change ≥ 1 relative to untreated controls was selected. The genes are listed in Additional file 4: Table S2.
