# Supplementary material for: The c-Myc-regulated lncRNA NEAT1 and paraspeckles modulate imatinib-induced apoptosis in CML cells
Source: Mol Cancer. 2018 Aug 28;17:130. doi: 10.1186/s12943-018-0884-z (PMC6114538; doi:10.1186/s12943-018-0884-z)
Supplement: Supplementary file 3 — Materials and methods. (DOCX 19 kb) [file 12943_2018_884_MOESM3_ESM.docx]

**Methods and Materials.**

**Samples**

Peripheral blood were obtained from patients with CML and from healthy adult donors in the First Affiliated Hospital of Jinan University. Twenty-six patients with Philadelphia chromosome (Ph1) positive CML were examined. The median age was 37 years (range: 17–79 years). Cytogenetic and molecular analyses were performed at diagnosis. Mononuclear cells were isolated by Histopaque gradient. All of the procedures were conducted according to the guidelines of the Medical Ethics Committee of the Health Bureau of the Guangdong Province of China. This study was approved by the Ethics Committee of First Affiliated Hospital of Jinan University, Guangzhou, China.

**Cell lines and cell cultures**

K562, NB4, Molt4, HL60, and Jurkat cells were cultured in RPMI 1640 medium (HyClone, SH30027) containing 10% fetal bovine serum (HyClone, SV30160) at 37°C in a 5% CO_2_ incubator. IM was purchased from Sigma-Aldrich and used at a final concentration of 1 μM.

**Quantitative real-time PCR analysis**

Total RNA was isolated using the Trizol Reagent (Life Technologies) and treated with DNase I. RNA was reverse transcribed using the High-Capacity cDNA reverse transcription kit (Applied Biosystems). qRT-PCR was performed to detect the expression of lncRNA and mRNA. ATCB served as internal control. The qRT-PCR cycling program was as follows: 95°C for 15 min followed by 40 cycles at 95°C for 10 s and 60°C for 30 s. The primers used were as follows: NEAT1 forward: 5’-CTTCCTCCCTTTAACTTATCCATTCAC-3’ and reverse: 5’-CTCTTCCTCCACCATTACCAACAATAC-3’ and NEAT1_2 forward: 5’- CAGTTAGTTTATCAGTTCTCCCATCCA-3’ and reverse: 5’-GTTGTTGTCGTCACCTTTCAACTCT -3’.

**Transfection**

K562 cells were transfected using the Neon® Transfection System (Invitrogen) with 100 pmol of oligonucleotides in 10 μl reactions. Transfections were performed as described previously. The NEAT1 siRNA sequences were as follows: si-NEAT1-1: 5’- GTGAGAAGTTGCTTAGAAACTTTCC-3’, si-NEAT1_2-1: 5’- GGGTAAATCTCAATCTTAA-3’, si-NEAT1-2: 5’- CTGGTATGTTGCTCTGTATGGTAAG-3’, and si-NEAT1_2-2: 5’- GGAACATTCTCATTTAATA -3’. BCR-ABL-specific sequences were 5’-GCAGAGTTCAAAAGCCCTT-3’ (si-BCR-ABL-1) and 5’-GAGTTCAAAAGCCCTTCAG -3’ (si-BCR-ABL-2),

**Luciferase assay**

K562 cells were transfected with the pGL3-promoter empty vector or a pGL3-promoter vector encoding a 1,989-bp DNA fragment from upstream of the NEAT1 gene. Quantification of all luciferase activities was performed with a dual-luciferase reporter assay system (Promega). Activities were normalized by co-transfection with the control thymidine kinase-driven Renilla luciferase plasmid phRL-TK (Promega).

**ChIP assay**

ChIP was performed as described previously. The real-time PCR (for quantification of ChIP assay) conditions were as follows: 95°C for 15 min followed by 40 cycles at 95°C for 10 s and 60°C for 20 s. Ct values for each sample were normalized to the Ct value obtained from each PCR reaction using the corresponding input genomic DNA as template. Primer sequences were as follows: primer A forward (-77/-59): 5’-AGGAATCGTCCCGTTGAGC-3’ and reverse (+23/+31): 5’-CTACACCCAGGCGCGC-3’; primer B forward (+23/+31): (5’-GCGCGCCTGGGTGTAG-3’) and reverse (+120/+133): (5’-CTCGCACCCCCAGCC-3’); and primer C forward (-299/-281): (5’-CGCCCGACCTCAACAACAT-3’) and reverse (-219/-202): (5’-CTTTTTGGGATCGCGGAC-3’); primer NC forward: 5’-AACAGGGGGTGCCAGTAAC-3’ and reverse: 5’-TCTGATGGTTGATGAAGCAAGGG.

**Statistical analysis**

Data were expressed as the mean ± SD of 3 independent experiments. The significance of the differences between groups was determined by a two-tailed Student t test. A P-value <0.05 was considered significant.
